# PI3K over-expression drives canine mammary tumor progression

**DOI:** 10.3389/fvets.2026.1897100

**Published:** 2026-08-05

**Authors:** Rong Chen, Shipeng Liu, Junxin Li, Xiaowei Huang, Yana Li, Xiaohong Huang, Zhaoyan Lin

**Affiliations:** 1 College of Animal Science, Fujian Agriculture and Forestry University, Fuzhou, China; 2 University Key Laboratory for Integrated Chinese Traditional and Western Veterinary Medicine and Animal Healthcare in Fujian Province, Fuzhou, China; 3 Fujian Key Laboratory of Traditional Chinese Veterinary Medicine and Animal Health, Fujian Agriculture and Forestry University, Fuzhou, China

**Keywords:** apoptosis, canine mammary tumor, over-expression, PI3K/AKT pathway, xenograft mouse model

## Abstract

Canine mammary tumor (CMT) represents the most frequently diagnosed neoplasm in intact female dogs and exhibits a high malignant rate. The PI3K/AKT pathway critically drives tumor progression, and serves as a target for multiple anti-tumor drugs. In this study, two CMT cell lines (CHMp and CIPm) were employed to assess the impact of lentivirus-mediated PI3K over-expression. Functional assays were performed to evaluate proliferation, migration, and invasion capacities, as well as the expression levels of associated proteins. As a result, PI3K over-expressing cells exhibited a significantly increase in proliferation, migration, and invasion compared to cells in negative control (NC) group. Additionally, western blot analyses revealed that PI3K over-expression evoked concordant alterations in apoptosis-related proteins, including elevated phospho-AKT and Bcl-2 coupled with reduced Bax, implying that PI3K-mediated attenuation of apoptosis facilitated tumor progression. Moreover, results from tumor xenograft mouse model experiments indicated that PI3K over-expression significantly promotes tumor growth *in vivo*. The present study confirmed the function of PI3K in accelerating the growth and invasive capacity of CMT, laying an experimental foundation for the exploration of PI3K-targeted therapies.

## 1 Introduction

Canine mammary tumor (CMT) is the most common neoplasms in intact female dog (1, 2), with malignant lesions detected in up to 83.3% of clinical surgical cases (3). The onset, progression and malignant transformation of CMTs are regulated by diverse risk factors, while oncogenic signaling cascades are closely associated with their malignant potential, invasive capacity and clinical prognosis. Aberrant phosphatidylinositol-3-kinase/protein kinase B (PI3K/AKT) signaling facilitates CMT proliferation, epithelial-mesenchymal transition (EMT), anti-apoptosis, lymphatic invasion and distant metastasis (4). The mitogen-activated protein/extracellular signal-regulated kinase kinase (MEK) and extracellular signal-regulated kinase (ERK) are both expressed and activated in CMT cell lines and tumor biopsy tissues (5). Meanwhile, CMT cell lines with high Wnt activity exhibit morphological and gene expression profiles consistent with stemness characteristics (6). Elucidating the molecular mechanisms underlying CMT initiation and progression is fundamental for precise prognostication and for the development of targeted therapeutics in affected dogs. Notably, the histological and molecular similarities between CMT and human breast cancers extend to hormone receptor expression patterns and metastatic behavior (7, 8), making dogs exceptional natural models for studying human mammary tumor progression.

The PI3K/AKT signaling pathway has emerged as a central regulator of canine oncogenic processes (9, 10). This highly conserved signaling cascade integrates multiple extracellular stimuli to coordinate fundamental cellular processes including growth, metabolism, and survival (9, 11). Class I PI3K is activated by receptor tyrosine kinases (RTKs) via two modes, then generates phosphatidylinositol-3,4,5-triphosphate (PIP3) to recruit and activate AKT, while phosphatase and tensin homolog (PTEN) reverses this lipid conversion to inhibit AKT signaling (12). Dysregulation of the PI3K/AKT signaling cascade represents a ubiquitous oncogenic driver event across diverse human and mammalian tumor types (13), and several inhibitors targeting major components of this pathway have been supported by the FDA (14).

In canine and human breast cancers, hyperactivation of PI3K signaling due to mutations or amplification is strongly associated with tumor proliferation, survival, metabolic reprogramming and therapeutic resistance (15–18). A study has demonstrated that elevated p-AKT levels in CMT specimens correlate with highly aggressive tumor subtypes, lymphovascular invasion and unfavorable survival outcomes (19). Recent reports have further delineated the PI3K pathway’s role in tumor microenvironment remodeling (20, 21) and immune evasion (22, 23), highlighting its multifaceted contributions to cancer progression. Therapeutic strategies targeting the PI3K/AKT signaling axis can also effectively suppress the progression of CMT (24, 25).

Beyond its established roles in cell survival and proliferation, the PI3K/AKT signaling pathway exerts precise control over apoptotic regulation through direct modulation of Bcl-2 family proteins (26, 27). Meanwhile, Bax is regulated by the phosphorylation of AKT, which inhibits its translocation to the mitochondria by maintaining the protein in the cytoplasm and promoting heterodimerization with antiapoptotic Bcl-2 family members (28–30). Moreover, the phosphorylation of AKT has been shown to effectively switch Bax from a pro-apoptotic to an anti-apoptotic role by preventing its mitochondrial insertion (31). Owing to their proliferative property, the central regions of solid tumors often undergo apoptosis and tissue necrosis resulting from ischemia and hypoxia (32). Aberrant PI3K activation confers potent anti-apoptotic capacity on neoplastic cells, which allows tumor cells to overcome such restrictive microenvironmental stress and boost tumor growth (32, 33).

Although existing studies have confirmed the widespread dysregulation of the PI3K signaling pathway in CMT, the specific biological functions mediated by PI3K over-expression in CMT cells remain to be characterized. In present study, two CMT cell lines were used to conduct PI3K over-expression via lentiviral transduction, the effects of PI3K over-expression were validated *in vitro* and *in vivo*, while apoptosis-related mediators were concurrently profiled to elucidate the underlying mechanism. The results of the present study provide a theoretical reference for developing therapeutic strategies targeting the PI3K signaling pathway against CMT.

## 2 Materials and methods

### 2.1 Cell lines and cell culture

The CMT cell lines (CHMp and CIPm, Graduate School of Agricultural and Life Sciences, University of Tokyo, Tokyo, Japan) (34) were cultured in Dulbecco’s Modified Eagle Medium (DMEM, Life Technologies Inc., Carlsbad, CA, USA) with 10% fetal bovine serum (FBS, Life Technologies Inc., CA USA), penicillin (100 units/mL) and streptomycin (0.1 mg/mL) (Life Technologies Inc., CA, USA). For lentivirus-transduced cells, 2 μg/mL puromycin (ST551, Beyotime, Shanghai, China) was supplemented to the culture medium for the screening of stably transfected cell lines. Cell lines were cultured at 37 °C in an atmosphere containing 5% CO_2_. Cells were passaged when reaching 80–90% confluence and seeded into culture plates of different specifications according to the requirements of distinct assays.

### 2.2 Lentiviral transduction

The PIK3CB-encoding lentiviral backbone (pLVX-AcGFP1-N1) was procured from OLIGOBIO Co., Ltd. (Beijing, China). For viral particle production, the rLv-PIK3CB overexpression (OE) construct and its corresponding negative control (NC) were, respectively, co-transfected with the psPAX2 and pMD2. G packaging plasmids into CHMp and CIPm cells using Lipofectamine 2000 (Life Technologies, CA, USA). The green fluorescent protein (GFP) signal was subsequently visualized with a confocal laser-scanning microscope (STELLARIS 5, Leica, Wetzlar, Germany). Lentivirus-transduced cells were screened with puromycin, and transduction efficiency was verified via immunofluorescence and western blot assays.

### 2.3 Immunofluorescence (IF) assay

CHMp and CIPm cells (5 × 10^4^ cells/mL) grown on cell culture coverslips were fixed with 4% paraformaldehyde for 15 min at room temperature, permeabilized with 0.1% Triton X-100 for 10 min, and blocked with 5% BSA for 30 min. Primary antibodies (rabbit anti-PI3K-p110β, 1:200. Proteintech Group, Inc. Wuhan, China) were applied overnight at 4 °C. After washing with PBS, cells were incubated with Alexa Fluor 488-conjugated goat anti-rabbit IgG (1:500. Proteintech Group, Inc. Wuhan, China) for 1 h at room temperature in the dark. Then, the coverslips were mounted on slides with DAPI (S2110, Solarbio, Beijing, China). The slides were visualized and images were taken with a confocal microscope (STELLARIS 5, Leica Inc., Wetzlar, Germany). Three sets of photographs were taken for each group and the experiments were carried out in triplicate.

### 2.4 Western blot assay

Protein was extracted from harvested CHMp and CIPm cells using RIPA buffer (Beyotime, Shanghai, China) and quantified by bicinchoninic acid (BCA) analysis (Life Technologies Inc., CA, USA). Equal amounts of protein were separated by 10% SDS-PAGE and transferred to PVDF membranes. After blocking with 5% non-fat milk for 1 h at room temperature, membranes were incubated overnight at 4 °C with primary antibodies (rabbit anti-PI3K-p110β, 1:1000, 20,584-1-AP, Proteintech; rabbit anti-AKT, 1:5000, CPA1034, Cohesion Biosciences; rabbit anti-pAKT, 1:5000, CPA1031, Cohesion Biosciences; rabbit anti-Bcl-2, 1:5000, CPA1095, Cohesion Biosciences; rabbit anti-Bax, 1:5000, CPA1091, Cohesion Biosciences; mouse anti-*β*-actin, 1:5000, 20,536-1-AP, Proteintech). Then membranes were incubated with corresponding HRP-conjugated secondary antibodies (goat anti-rabbit IgG, 1:5000, SA00001-9, Proteintech; goat anti-mouse IgG, 1:5000, SA00001-1, Proteintech) for 1 h at room temperature, followed by immersion in chemiluminescence working solution for 30 s, and exposed under chemiluminescent imaging analysis system (MicroChemi4.2, DNR Bio Imaging Systems, Isreal). Densitometry analysis was conducted using ImageJ (Version 1.52i, National Institutes of Health, USA) software. The experiments were carried out in triplicate.

### 2.5 Cell viability assay

Cell viability was detected using Cell Counting Kit 8 (CCK-8, GLPBIO, CA, USA) according to the manufacturer’s protocol. Briefly, CHMp and CIPm cells were seeded in 96-well plates at a density of 1 × 10^3^ cells/well and cultured at 37 °C for 24h. Then, 10 μL of CCK-8 reagent was added to each well, followed by incubation at 37 °C for 2 h. The absorbance at 450 nm was measured using a microplate reader (Molecular Devices, 22,202-SANGMSMA1, Shanghai, China). The mean optical density (OD, absorbance) of five wells in the indicated groups was used to calculate the percentage of cell viability as follows: percentage of cell viability = A_treatment_/A_control_ × 100% (where A = absorbance). The average absorbance of the wild type (WT) group was set to 100%. The experiments were carried out in triplicate.

### 2.6 Cell migration assay

Cell migratory capacity was evaluated using the wound healing assay (35). Briefly, CHMp and CIPm cells were seeded in 6-well plates and cultured until reaching 90–100% confluence. A sterile 200 μL pipette tip was used to create a straight scratch wound in the monolayer. Detached cells were removed by washing twice with PBS, and fresh medium was added. The width of the scratch was observed and measured using a microscope (IRX50, Sunny Optical Technology Inc., Jiangsu, China) immediately after washing and following 24 h of cell incubation. The migration rate was calculated as: Migration rate (%) = [(Wound width at 0 h − Wound width at 24 h) / Wound width at 0 h] × 100%. The experiments were carried out in triplicate.

### 2.7 Cell invasion assay

Cell invasion ability was evaluated using Transwell chambers (8 μm pore size; Corning, USA) pre-coated with Matrigel (BD Biosciences, USA). In brief, 5 × 10^4^ CHMp and CIPm cells suspended in serum-free medium were seeded into the upper chamber, while the lower chamber was filled with complete medium containing 10% FBS. After 24 h of incubation at 37 °C, non-invaded cells on the upper chamber surface were gently wiped off with sterile cotton swabs. Cells that had invaded to the lower surface were fixed with 4% paraformaldehyde for 15 min at room temperature, then stained with 0.1% crystal violet for 20 min at room temperature, and imaged under a microscope (IRX50, Sunny Optical Technology Inc., Jiangsu, China). The number of invaded cells was counted from five randomly selected fields per membrane using ImageJ software (NIH, USA). The experiments were carried out in triplicate.

### 2.8 Colony formation assay

Briefly, CHMp and CIPm cells were seeded in 6-well plates at a density of 500 cells/well and cultured at 37 °C in complete medium for 10 days. The medium was refreshed every 3 days. After incubation, cells were washed twice with PBS, fixed with 4% paraformaldehyde for 15 min at room temperature, and stained with 0.5% crystal violet (Solarbio, Beijing, China) for 30 min at room temperature. Colonies containing >50 cells were photographed and counted manually. The experiments were carried out in triplicate.

### 2.9 Flow cytometry assay

Cell apoptosis was quantified via Annexin V-FITC/PI double staining (KGA1013, KeyGEN Bio TECH, Nanjing, China) followed by flow cytometry. Briefly, CHMp and CIPm cells were digested, harvested and rinsed twice with pre-cooled PBS, then resuspended in Annexin V binding buffer at a density of 1 × 10^6^ cells/mL. A total of 100 μL cell suspension was transferred into flow tubes, mixed with 5 μL Annexin V-FITC and 5 μL PI working solution, and incubated for 15 min at room temperature in dark. After adding 400 μL binding buffer, samples were immediately analyzed on a flow cytometer (Agilent NovoCyte, USA). Unstained, single Annexin V-stained and single PI-stained cells were prepared as compensation controls to correct fluorescence spillover. For each sample, 20,000 cellular events were collected and recorded. The proportions of early apoptotic populations were calculated using FlowJo (Version 10, USA) software. The experiments were carried out in triplicate.

### 2.10 Tumor xenograft mouse mode

The animal study was reviewed and approved by the Animal Ethics Committee of Fujian Agriculture and Forestry University (approval code PZCASFAFU24131), according to the guidelines for Laboratory Animal Use and Care from the Chinese Center For Disease Control and Prevention and the Rules for Medical Laboratory Animals (1998) from the Chinese Ministry of Health.

Five-week-old female BALB/c nude mice (SLAC, Shanghai, China) were randomly divided into three groups: WT, NC and PI3K over-expression groups, with six mice per group (n = 6). Mice were housed in standard individually ventilated cages (IVCs) with automatic water bottles, and had ad libitum access to food and water. For tumor inoculation, 1 × 10^6^ CHMp cells in 150 μL PBS were subcutaneously injected into the right mammary fat pad of each mice. Tumor size was measured every 3 days using calipers, and volume was calculated as: 0.5 × Length × Width^2^. Mice were raised for 15 consecutive days. At the end of the experiment, all mice were sacrificed and xenograft tumors were excised and weighed. Tumor tissues were fixed in 4% paraformaldehyde, embedded in paraffin blocks for subsequent experiments.

### 2.11 Immunohistochemistry (IHC) analysis

Tissue sections (4 μm thick) were deparaffinized in xylene and rehydrated through a graded ethanol series. Antigen retrieval was performed by heating in citrate buffer (pH 6.0) at 95 °C for 15 min. Endogenous peroxidase activity was blocked with 3% hydrogen peroxide for 10 min at room temperature. After blocking with 5% normal goat serum for 30 min, sections were incubated overnight at 4 °C with primary antibodies (rabbit anti-PI3K-p11β, 1:200, 20,584-1-AP, Proteintech). The next day, sections were washed with PBS for 5 times and then incubated with biotinylated secondary antibodies for 1 h at room temperature. Diaminobenzidine (DAB) was used as the chromogen, followed by counterstaining with hematoxylin. Slides were dehydrated, cleared in xylene, and mounted with neutral balsam. Images were captured using a microscope (IRX50, Sunny Optical Technology Inc., Jiangsu, China). Three sets of photographs were taken for each group and the experiments were carried out in triplicate.

### 2.12 Deoxynucleotidyl transferase-mediated dUTP Nick end labelling (TUNEL) assay

The TUNEL assay was performed using One step TUNEL apoptosis detection kit (KGA1405-100, KeyGEN Bio TECH, Nanjing, China) according to the manufacturer’s protocols. Briefly, tissue sections (4 μm thick) were deparaffinized in xylene and permeabilized with 20 μg/mL Proteinase K at 37 °C for 20 min. Then the TUNEL working solution was evenly applied to each slice, followed by a incubation at 37 °C for 1 h in dark. After PBS washing, DAPI was used for nuclear counterstaining. Slides were mounted with anti-fade medium and images were captured with a confocal microscope (STELLARIS 5, Leica Inc., Wetzlar, Germany). Three sets of photographs were taken for each group and the experiments were carried out in triplicate.

### 2.13 Statistical analysis

Statistical analysis was performed using GraphPad Prism10 software (GraphPad Software Inc., San Diego, CA, USA). A two-tailed unpaired t-test with Welch’s correction was applied when the variances of two groups were proven equal by the F test. A *p* value of 0.05 or less indicated statistical significance.

## 3 Results

### 3.1 PI3K over-expression significantly enhances the proliferative capacity of CMT cells

**Figure 1:**
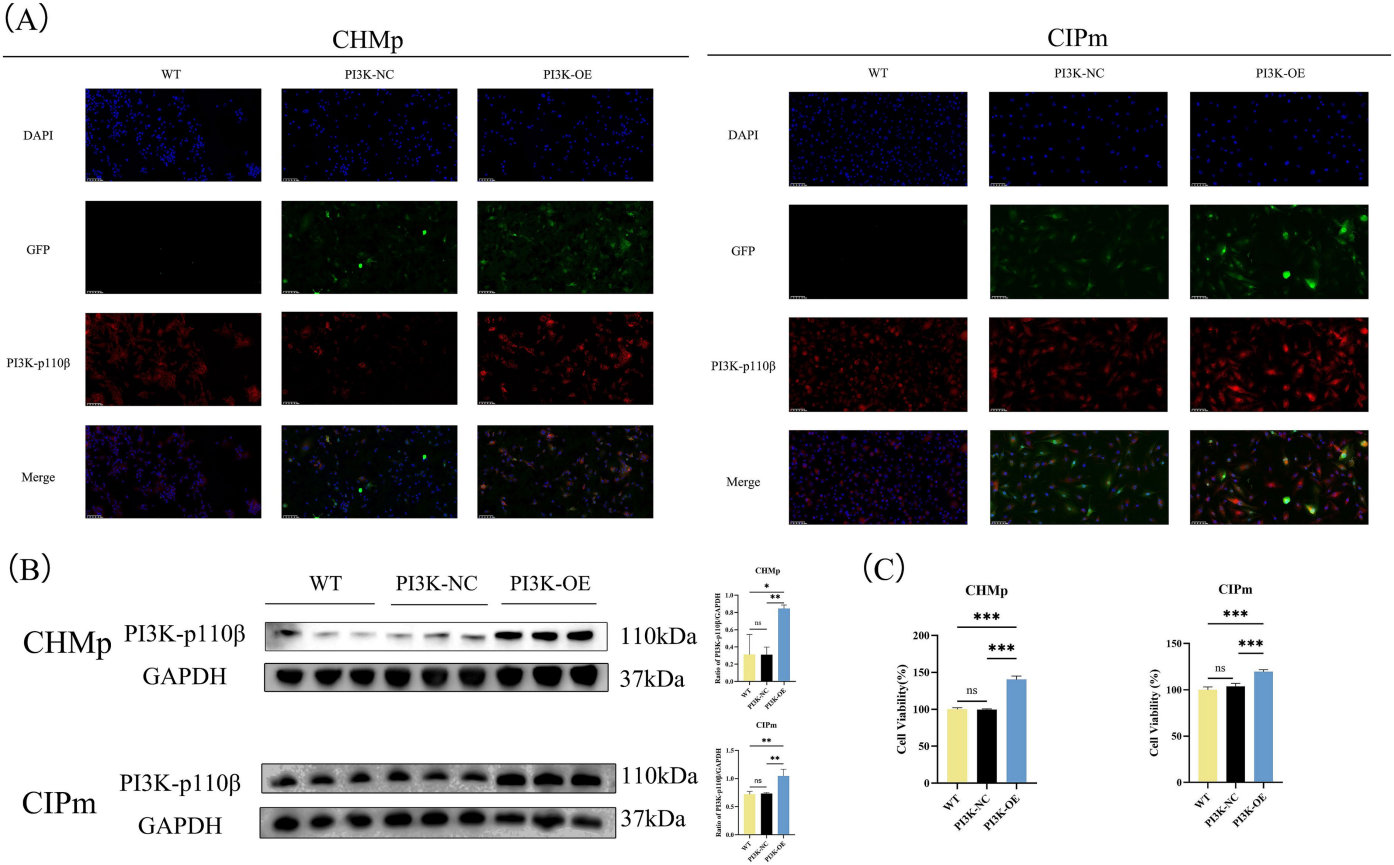
PI3K Overexpression Significantly Enhances the Proliferative Capacity of CMT Cells. **(A)** Immunofluorescence and **(B)** Western blot validation of lentivirus-mediated PI3K over-expression efficiency. **(C)** Cell viability determined by Cell Counting Kit-8 assay. Scale bar: 100 μm. *p < 0.05, **p < 0.01, ***p < 0.001. Abbreviations: WT, wild type; NC, negative control; OE, over-expression; GFP, green fluorescent protein; ns, non-significant.

To verify lentiviral transduction efficiency, immunofluorescence (IF) and Western blot analyses were performed in CHMp and CIPm cells. The GFP positivity in both the PI3K overexpression and NC groups confirmed successful lentiviral transduction; although PI3K protein expression was detectable in all three cell groups, the PI3K over-expression group exhibited significantly elevated PI3K protein levels compared with the WT and NC groups, verifying the successful establishment of the PI3K over-expressing cell modellevels (Figure 1A).

Western blot analysis further validated the intracellular PI3K expression levels, and the results were highly consistent with the IF observations. PI3K protein expression was significantly higher in the PI3K over-expression group than in the WT and NC groups (*p* < 0.05, Figure 1B).

Cell viability assays revealed no significant difference between WT and NC groups (*p* > 0.05). In contrast, cells with PI3K over-expression exhibited a remarkable increase in cell viability (*p* < 0.001), indicating that PI3K over-expression enhances the proliferative capacity of CMT cells (Figure 1C).

### 3.2 PI3K over-expression significantly enhances the migration, invasion and Colony formation capacities of CMT cells

**Figure 2:**
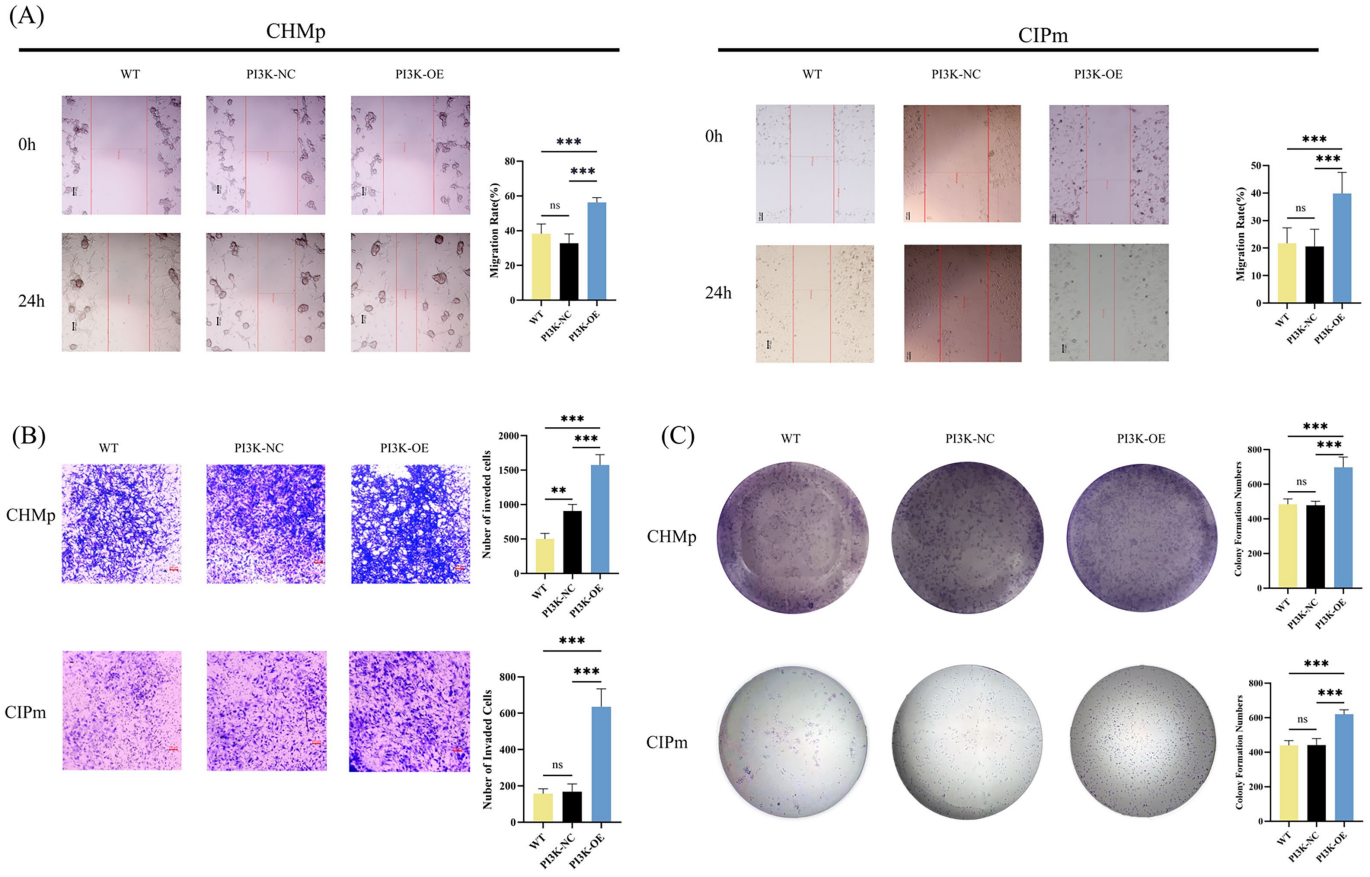
PI3K over-expression significantly enhances the migration, invasion and colony formation capacities of CMT cells. **(A)** Wound-healing assay for cell migration. **(B)** Matrigel-coated Transwell assay for invasion. **(C)** Colony-formation assay; 500 cells/well were seeded in 6-well plates and cultured in complete medium for 10 days. Scale bar: 100 μm. **p < 0.01, ***p < 0.001; Abbreviations: WT, wild type; NC, negative control; OE, over-expression; ns, non-significant.

In order to further investigate the influence of PI3K over-expression on CHMp and CIPm cells, cell migration assay, transwell invasion assay, and colony formation assay were performed. The results showed that there were no significant differences in cell migration capability between the WT and NC groups of both CMT cell lines (*p* > 0.05), while cells with PI3K over-expression exhibited remarkably stronger migration capability than the WT and NC groups (*p* < 0.001, Figure 2A).

Regarding the cell invasion assay, the NC group of CHMp cells presented higher invasive ability than the WT group (*p* < 0.05), whereas no significant difference was found between the WT and NC groups in CIPm cells. In both CMT cell lines, the PI3K over-expression groups possessed substantially more invasive cells compared with the WT and NC groups (*p* < 0.001, Figure 2B).

For clonogenicity, no statistically significant difference existed between the WT and NC groups in the two CMT cell lines (*p* > 0.05), the clonogenicity of PI3K over-expression cells was markedly higher than that of the WT and NC groups (*p* < 0.001, Figure 2C).

Taken together, these results indicated that PI3K over-expression specifically enhances the migratory, invasive and colony-forming capacity of CMT cells.

### 3.3 PI3K over-expression modulates the expression of apoptosis-related proteins in CMT cells

**Figure 3:**
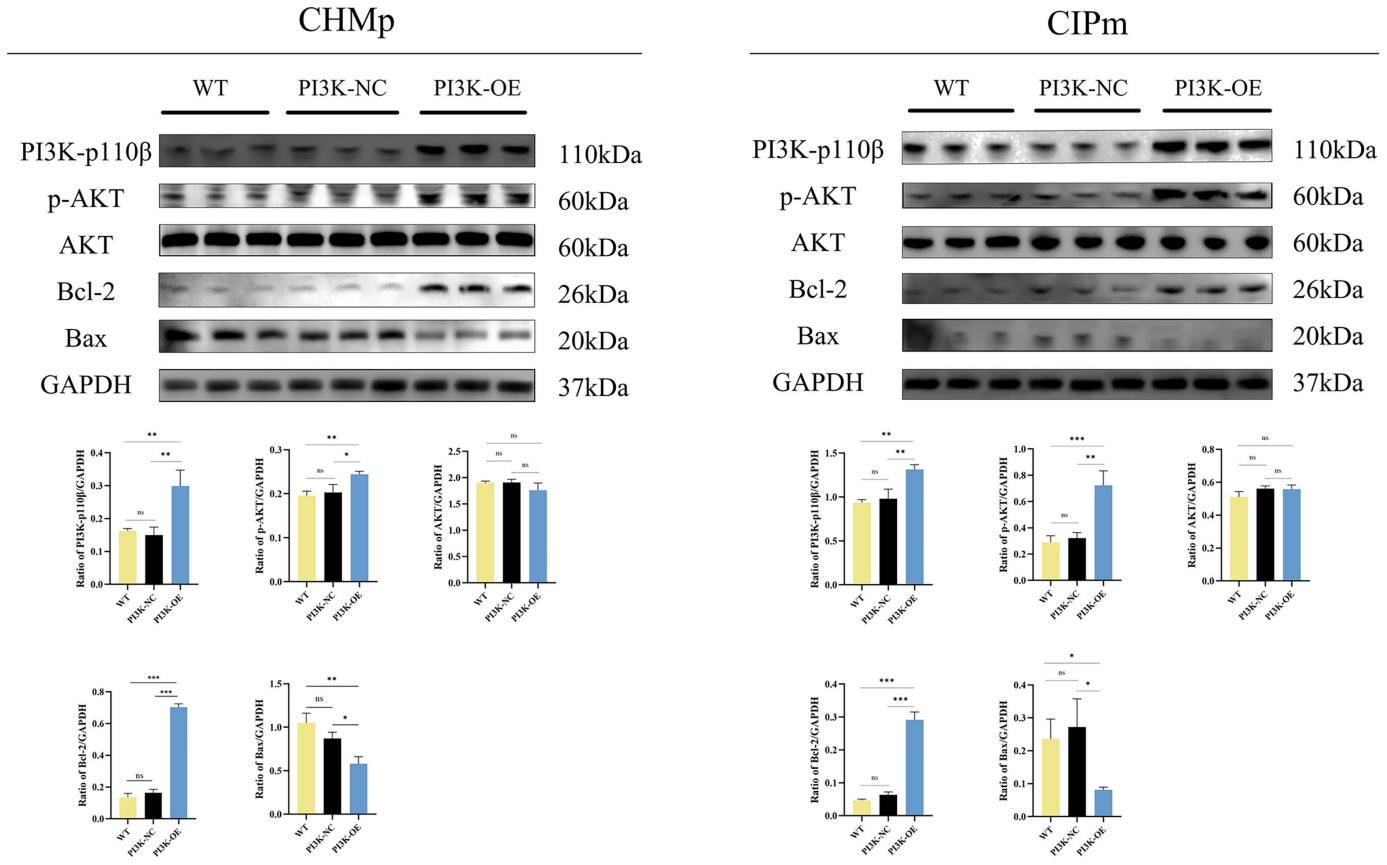
PI3K overexpression modulates the expression of apoptosis-related proteins in CMT cells. Western blot analysis of CHMp and CIPm cells. *p < 0.05, **p < 0.01, ***p < 0.001. Abbreviations: WT, wild type; NC, negative control; OE, overexpression; ns, non-significant.

In view of the established role of PI3K–AKT signaling in apoptotic regulation, the expression of key apoptotic regulators was examined by Western blot. In both CHMp and CIPm cells, there were no significant alterations in the expression levels of PI3K, phosphorylated AKT (p-AKT), Bcl-2 and Bax between WT and NC cells. Compared with the WT and NC groups, CMT cells with PI3K over-expression exhibited significantly upregulated PI3K, p-AKT and Bcl-2 (*p* < 0.05), accompanied by a notable decrease in Bax expression (*p* < 0.05). These data demonstrated that PI3K over-expression activates AKT signaling, upregulates anti-apoptotic proteins and inhibits pro-apoptotic pathways (Figure 3).

Meanwhile, the apoptotic rate of CMT cells was further measured by flow cytometry. However, the basal apoptotic rate of CMT cells was inherently low, and no significant difference in the proportion of apoptotic cells was observed among groups (*p* > 0.05, Supplementary Figure 1).

### 3.4 PI3K over-expression accelerates xenograft tumor growth

**Figure 4:**
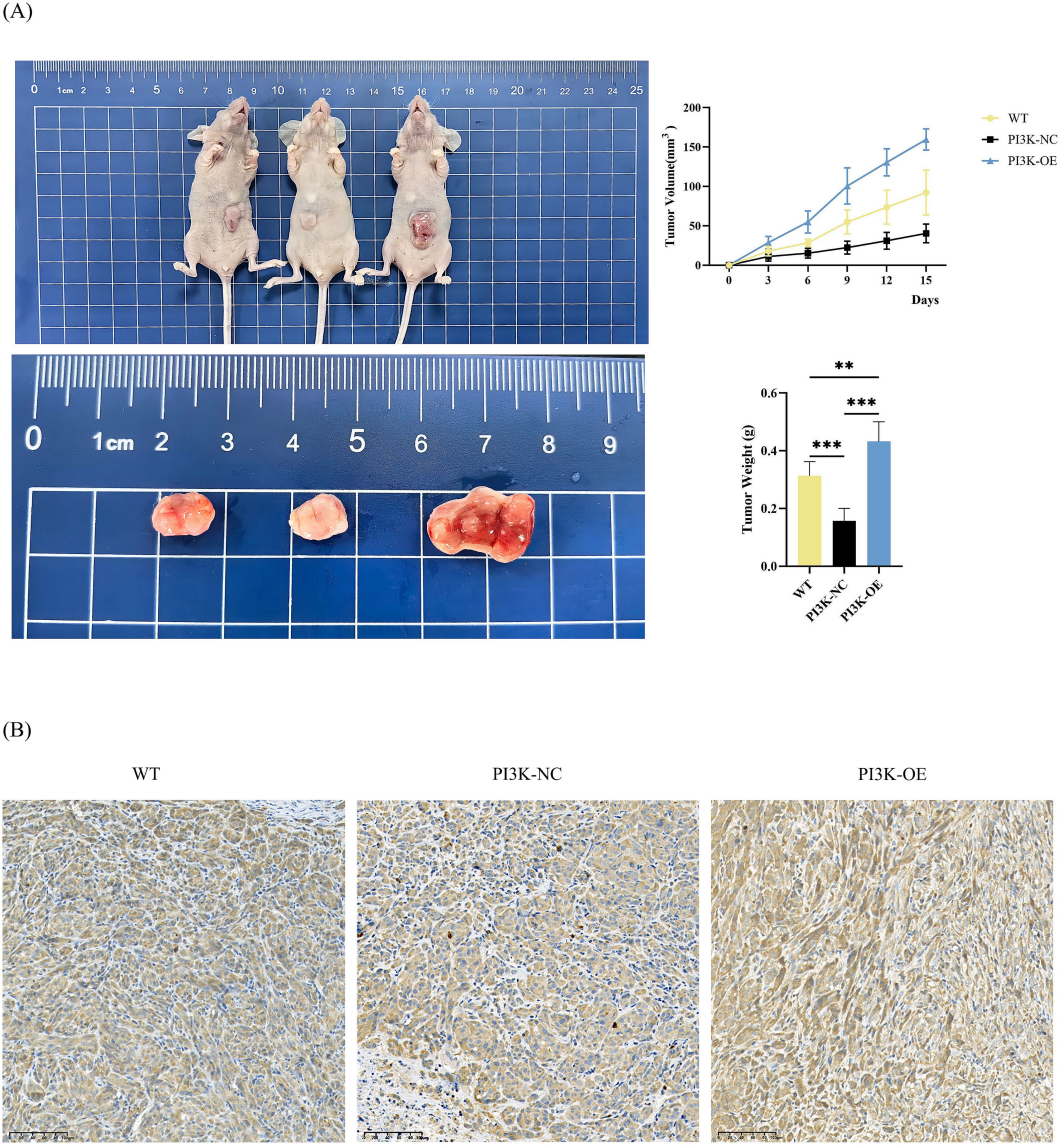
PI3K overexpression accelerates xenograft tumor growth. **(A)** Evaluation of tumor growth in the CHMp xenograft model. Tumor volume trends over time in mice and Final tumor weight. **(B)** IHC analysis of tumor tissues. Scale bar: 100 μm *p* < 0.05, **p* < 0.01, ***p* < 0.001. Abbreviations: WT, wild type; NC, negative control; OE, overexpression; ns, non-significant.

To validate the role of PI3K over-expression in CMT *in vivo*, a xenograft tumor model was established. As a result, after 15 days of feeding and tumor growth, the tumors derived from CHMp cells in the PI3K over-expression group were significantly larger and heavier than those in the WT and NC groups (*p* < 0.01, Figure 4A), indicating that PI3K over-expression promotes CMT growth *in vivo*, this result was consistent with the *in vitro* cell viability data presented in Figure 1, demonstrating that PI3K over-expression accelerates the growth of CMT cells both *in vitro* and *in vivo*. Conversely, tumor volume and weight in the NC group were markedly lower relative to the control group (*p* < 0.05, Figure 4A). Consistently, IHC analysis showed that the PI3K protein expression level in tumor tissues of the PI3K over-expression group was markedly higher than in the WT and NC groups (*p* < 0.001, Figure 4B). The mice were dissected, yet no obvious metastatic tumor lesions were detected in visceral organs. Consistent with the *in vitro* findings, TUNEL staining revealed no significant intergroup differences in the apoptotic levels within xenograft tumor tissues (Supplementary Figure 2).

## 4 Discussion

The PI3K/AKT axis operates as a central oncogenic driver that integrates cell survival, proliferation, metabolic reprogramming, and invasive capacity (36). Its sustained hyperactivation propels tumor progression by hijacking downstream executors: up-regulating cyclin D1 to expedite G1–S transition, tilting the Bcl-2/Bax balance toward anti-apoptosis, and inducing matrix metalloproteinases (MMPs) that dismantle extracellular barriers to invasion (37). PI3K activation has been documented to drive tumor progression in both canine (4, 24) and human (38) cancers, which is consistent with the present study. Moreover, PI3K over-expression can precipitate excessive tumor growth and chemo-resistance by suppressing apoptosis (39), a previous study has demonstrated that PI3K over-expression significantly attenuates the apoptotic effect of anti-CMT drugs on CMT cells (24), consistent with this viewpoint, the present study also confirmed that PI3K over-expression alters the expression of apoptosis related proteins in CMT cells (Figure 3), which suggests that PI3K expression levels may influence the efficacy of anti-apoptotic therapeutic strategies against CMT.

In the present study, PI3K over-expression was found to significantly promote the growth of CMT cells both *in vitro* and *in vivo*, which provides a reference for developing and applying anti-PI3K strategies against CMT. Although CMT cells with PI3K over-expression exhibited stronger invasive capacity than the WT and NC groups in the *in vitro* assays (Figure 2B), no metastatic tumor lesions were observed in the visceral organs of mice from all groups in the *in vivo* experiment. This phenomenon may be attributed to the short 15-day housing period that was insufficient for tumor metastasis to occur, or inherent biological characteristics of the cell lines used. Future experiments employing mouse metastasis models will help clarify whether PI3K over-expression accelerates the invasive and metastatic potential of CMT *in vivo*.

Significant alterations in multiple oncogenic phenotypes and anti-apoptotic properties were detected in CMT cells with PI3K over-expression relative to the WT and NC groups, nevertheless, significant differences between the WT and NC groups were observed in some experiments: in invasion assays using CHMp cells, the NC group displayed higher invasiveness than the WT group (Fiugure 2B), whereas in the xenograft tumor model, the NC group exhibited smaller and lighter tumors (Figure 4A). These discrepancies may be attributable to the empty-vector lentiviral transduction applied to the NC group, studies have demonstrated that lentiviral vectors can indeed exert multiple effects on tumor cells, including modulation of antitumor immunity (40), cellular apoptosis (41), and cellular sensitivity to chemotherapeutic agents (42), although the precise mechanisms underlying lentiviral vector mediated effects on CHMp cells remain to be fully elucidated. Importantly, the PI3K over-expression group differed significantly from both the NC and control groups, so these inter-control variations do not alter the overall conclusions of the study.

Results of the present study substantiate a causal role for PI3K in driving CMT growth and invasiveness, yet deeper mechanistic insights remain to be uncovered. Transcriptomic and proteomic profiling of PI3K over-expressing cell lines is required to map the network of related transcriptional and translational alterations. Although this study revealed that PI3K over-expression upregulates anti-apoptotic proteins in CMT cells (Figure 3), no significant intergroup differences in overall apoptotic rates were detected (Supplementary Figures 1, 2). This phenomenon is attributable to the inherently low basal apoptosis level of CMT cells. Distinct responses to pro-apoptotic agents may exist among different cell groups, and the effects of conventional chemotherapeutic drugs on these cells deserve further exploration in future research.

Meanwhile, this study lacks clinical specimens and survival/prognostic analyses. To clarify the correlation between PI3K and the histological grade of CMT, and to evaluate whether PI3K can serve as a surrogate biomarker for evaluating malignancy and prognosis in CMT patients, further investigations are required to determine the efficacy of conventional CMT therapies in patients with PI3K over-activated status and verify whether PI3K can act as a prognostic biomarker for chemotherapy and targeted therapy responses. Collectively, additional clinical research is warranted to fully elucidate the clinical relevance and translational value of PI3K in CMT.

## Data Availability

The original contributions presented in the study are included in the article/Supplementary material, further inquiries can be directed to the corresponding author/s.

## References

[1] Rodríguez J Santana Á Herráez P Killick DR de Los Monteros AE. Epidemiology of canine mammary tumours on the canary archipelago in Spain. BMC Vet Res. (2022) 18:268. doi: 10.1186/s12917-022-03363-9, 35821034 PMC9277912

[2] Zheng HH Du CT Yu C Zhang YZ Huang RL Tang XY . Epidemiological investigation of canine mammary tumors in mainland China between 2017 and 2021. Front Vet Sci. (2022) 9:843390. doi: 10.3389/fvets.2022.843390, 35812867 PMC9257276

[3] Dolka I Czopowicz M Stopka D Wojtkowska A Kaszak I Sapierzyński R. Risk factor analysis and clinicopathological characteristics of female dogs with mammary tumours from a single-center retrospective study in Poland. Sci Rep. (2024) 14:5569. doi: 10.1038/s41598-024-56194-z, 38448646 PMC10917774

[4] Kim SH Seung BJ Cho SH Lim HY Bae MK Sur JH. Dysregulation of PI3K/Akt/PTEN pathway in canine mammary tumor. Animals. (2021) 11:2079. doi: 10.3390/ani11072079, 34359206 PMC8300234

[5] Altamura G Uberti BD Galiero G Martano M Pirro A Russo M . Expression and activation of platelet-derived growth factor β receptor, mitogen-activated protein/extracellular signal-regulated kinase kinase (MEK) and extracellular signal-regulated kinase (ERK) in canine mammary tumours. Res Vet Sci. (2017) 110:29–33. doi: 10.1016/j.rvsc.2016.10.014, 28159233

[6] Timmermans-Sprang E Collin R Henkes A Philipsen M Mol JA. P-cadherin mutations are associated with high basal Wnt activity and stemness in canine mammary tumor cell lines. Oncotarget. (2019) 10:2930–46. doi: 10.18632/oncotarget.26873, 31105876 PMC6508207

[7] Bhutta ZA Choi KC. Canine mammary tumors as a promising adjunct preclinical model for human breast cancer research: similarities, opportunities, and challenges. Arch Pharm Res. (2025) 48:43–61. doi: 10.1007/s12272-024-01524-y, 39752109

[8] Kwon JY Moskwa N Kang W Fan TM Lee C. Canine as a comparative and translational model for human mammary tumor. J Breast Cancer. (2023) 26:1–13. doi: 10.4048/jbc.2023.26.e4, 36762784 PMC9981990

[9] Meuten TK Dean GA Thamm DH. Review: the PI3K-AKT-mTOR signal transduction pathway in canine cancer. Vet Pathol. (2024) 61:339–56. doi: 10.1177/03009858231207021, 37905509

[10] Miyanishi K Nururrozi A Igase M Tanabe M Sakurai M Sakai Y . Activation of the Akt signalling pathway as a prognostic indicator in canine soft tissue sarcoma. J Comp Pathol. (2023) 206:44–52. doi: 10.1016/j.jcpa.2023.08.007, 37839309

[11] Hoxhaj G Manning BD. The PI3K-AKT network at the interface of oncogenic signalling and cancer metabolism. Nat Rev Cancer. (2020) 20:74–88. doi: 10.1038/s41568-019-0216-7, 31686003 PMC7314312

[12] Chen YT Tan KA Pang LY Argyle DJ. The class I PI3K/Akt pathway is critical for cancer cell survival in dogs and offers an opportunity for therapeutic intervention. BMC Vet Res. (2012) 8:73. doi: 10.1186/1746-6148-8-73, 22647622 PMC3515332

[13] Jiang M Zhang K Zhang Z Zeng X Huang Z Qin P . PI3K/AKT/mTOR Axis in Cancer: from pathogenesis to treatment. MedComm. (2025) 6:e70295. doi: 10.1002/mco2.70295, 40740483 PMC12308072

[14] Li Q Li Z Luo T Shi H. Targeting the PI3K/AKT/mTOR and RAF/MEK/ERK pathways for cancer therapy. Molecular Biomedicine. (2022) 3:47. doi: 10.1186/s43556-022-00110-2, 36539659 PMC9768098

[15] Liu R Chen Y Liu G Li C Song Y Cao Z . PI3K/AKT pathway as a key link modulates the multidrug resistance of cancers. Cell Death Dis. (2020) 11:797. doi: 10.1038/s41419-020-02998-6, 32973135 PMC7515865

[16] Mei C Liu Y Liu Z Zhi Y Jiang Z Lyu X . Dysregulated signaling pathways in canine mammary tumor and human triple negative breast Cancer: advances and potential therapeutic targets. Int J Mol Sci. (2024) 26:145. doi: 10.3390/ijms26010145, 39796003 PMC11720488

[17] Noorolyai S Shajari N Baghbani E Sadreddini S Baradaran B. The relation between PI3K/AKT signalling pathway and cancer. Gene. (2019) 698:120–8. doi: 10.1016/j.gene.2019.02.076, 30849534

[18] Omolekan TO Chamcheu JC Buerger C Huang S. PI3K/AKT/mTOR signaling network in human health and diseases. Cells. (2024) 13:1500. doi: 10.3390/cells13171500, 39273070 PMC11394329

[19] Asproni P Millanta F Ressel L Podestà F Parisi F Vannozzi I . An Immunohistochemical study of the PTEN/AKT pathway involvement in canine and feline mammary tumors. Animals. (2021) 11:365. doi: 10.3390/ani11020365, 33535663 PMC7912927

[20] Li M Li M Yang Y Liu Y Xie H Yu Q . Remodeling tumor immune microenvironment via targeted blockade of PI3K-γ and CSF-1/CSF-1R pathways in tumor associated macrophages for pancreatic cancer therapy. J Control Release. (2020) 321:23–35. doi: 10.1016/j.jconrel.2020.02.011, 32035193

[21] Niu X You Q Hou K Tian Y Wei P Zhu Y . Autophagy in cancer development, immune evasion, and drug resistance. Drug Resist Updat. (2025) 78:101170. doi: 10.1016/j.drup.2024.101170, 39603146

[22] Collins NB Al Abosy R Miller BC Bi K Zhao Q Quigley M . PI3K activation allows immune evasion by promoting an inhibitory myeloid tumor microenvironment. J Immunother Cancer. (2022) 10:e003402. doi: 10.1136/jitc-2021-003402, 35264433 PMC8915320

[23] Zheng X Chen J Deng M Ning K Peng Y Liu Z . G3BP1 and SLU7 jointly promote immune evasion by downregulating MHC-I via PI3K/Akt activation in bladder Cancer. Adv Sci. (2024) 11:e2305922. doi: 10.1002/advs.202305922, 38084438 PMC10870071

[24] Lin Z Chen R Wang J Zheng Y He Z Yan Y . Auranofin suppresses the growth of canine mammary tumour cells and induces apoptosis via the PI3K/AKT pathway. Vet Comp Oncol. (2024) 22:555–65. doi: 10.1111/vco.13005, 39221701

[25] Park SY Baek YB Lee CH Kim HJ Kim HP Jeon YJ . Establishment of canine mammary gland tumor cell lines harboring PI3K/Akt activation as a therapeutic target. BMC Vet Res. (2024) 20:233. doi: 10.1186/s12917-024-04085-w, 38807154 PMC11134682

[26] Jiang Y Fang B Xu B Chen L. The RAS-PI3K-AKT-NF-κB pathway transcriptionally regulates the expression of BCL2 family and IAP family genes and inhibits apoptosis in fibrous epulis. J Clin Lab Anal. (2020) 34:e23102. doi: 10.1002/jcla.23102, 31743516 PMC7083487

[27] Korshunova AY Blagonravov ML Neborak EV Syatkin SP Sklifasovskaya AP Semyatov SM . BCL2-regulated apoptotic process in myocardial ischemia-reperfusion injury (review). Int J Mol Med. (2021) 47:23–36. doi: 10.3892/ijmm.2020.4781, 33155658 PMC7723511

[28] Gardai SJ Hildeman DA Frankel SK Whitlock BB Frasch SC Borregaard N . Phosphorylation of Bax Ser184 by Akt regulates its activity and apoptosis in neutrophils. J Biol Chem. (2004) 279:21085–95. doi: 10.1074/jbc.M400063200, 14766748

[29] Simonyan L Renault TT Novais MJ Sousa MJ Côrte-Real M Camougrand N . Regulation of Bax/mitochondria interaction by AKT. FEBS Lett. (2016) 590:13–21. doi: 10.1002/1873-3468.12030, 26763134

[30] Xin M Deng X. Nicotine inactivation of the proapoptotic function of Bax through phosphorylation. J Biol Chem. (2005) 280:10781–9. doi: 10.1074/jbc.M500084200, 15642728

[31] Kale J Kutuk O Brito GC Andrews TS Leber B Letai A . Phosphorylation switches Bax from promoting to inhibiting apoptosis thereby increasing drug resistance. EMBO Rep. (2018) 19:e45235. doi: 10.15252/embr.201745235, 29987135 PMC6123645

[32] Kim SY Tang M Chih SY Sallavanti J Gao Y Qiu Z . Involvement of p38 MAPK and MAPKAPK2 in promoting cell death and the inflammatory response to ischemic stress associated with necrotic glioblastoma. Cell Death Dis. (2025) 16:12. doi: 10.1038/s41419-025-07335-3, 39805854 PMC11729867

[33] Yang Q Xi Y Huang X An W Sun C Wang Q . Hypoxia-induced S100A10 promotes glioblastoma malignancy and chemoresistance by activating PI3K-AKT signaling pathway. Funct Integr Genomics. (2025) 25:202. doi: 10.1007/s10142-025-01693-z, 41026238

[34] Nakagawa T Watanabe M Ohashi E Uyama R Takauji S Mochizuki M . Cyclopedic protein expression analysis of cultured canine mammary gland adenocarcinoma cells from six tumours. Res Vet Sci. (2006) 80:317–23. doi: 10.1016/j.rvsc.2005.07.011, 16181651

[35] Grada A Otero-Vinas M Prieto-Castrillo F Obagi Z Falanga V. Research techniques made simple: analysis of collective cell migration using the wound healing assay. J Invest Dermatol. (2017) 137:e11–6. doi: 10.1016/j.jid.2016.11.020, 28110712

[36] Thorpe LM Yuzugullu H Zhao JJ. PI3K in cancer: divergent roles of isoforms, modes of activation and therapeutic targeting. Nat Rev Cancer. (2015) 15:7–24. doi: 10.1038/nrc3860, 25533673 PMC4384662

[37] Rascio F Spadaccino F Rocchetti MT Castellano G Stallone G Netti GS . The pathogenic role of PI3K/AKT pathway in Cancer onset and drug resistance. An Updated Review Cancers. (2021) 13:3949. doi: 10.3390/cancers13163949, 34439105 PMC8394096

[38] Yu L Wei J Liu P. Attacking the PI3K/Akt/mTOR signaling pathway for targeted therapeutic treatment in human cancer. Semin Cancer Biol. (2022) 85:69–94. doi: 10.1016/j.semcancer.2021.06.019, 34175443

[39] Glaviano A Foo ASC Lam HY Yap KCH Jacot W Jones RH . PI3K/AKT/mTOR signaling transduction pathway and targeted therapies in cancer. Mol Cancer. (2023) 22:138. doi: 10.1186/s12943-023-01827-6, 37596643 PMC10436543

[40] Bhuniya A Pattarayan D Yang D. Lentiviral vector transduction provides nonspecific immunogenicity for syngeneic tumor models. Mol Carcinog. (2022) 61:1073–81. doi: 10.1002/mc.23467, 36161729 PMC9643670

[41] Ha T DiPrima M Koparde V Jailwala P Ohnuki H Feng JX . Antisense transcription from lentiviral gene targeting linked to an integrated stress response in colorectal cancer cells. Molecular Therapy Nucleic Acids. (2022) 28:877–91. doi: 10.1016/j.omtn.2022.05.029, 35694213 PMC9163427

[42] Ranjbar B Krogh LB Laursen MB Primo MN Marques SC Dybkær K . Anti-apoptotic effects of lentiviral vector transduction promote increased rituximab tolerance in cancerous B-cells. PLoS One. (2016) 11:e0153069. doi: 10.1371/journal.pone.0153069, 27045839 PMC4821607

